# Multidrug-Resistant Infections and Metabolic Syndrome: An Overlooked Bidirectional Relationship

**DOI:** 10.3390/biomedicines13061343

**Published:** 2025-05-30

**Authors:** Carlo Acierno, Riccardo Nevola, Fannia Barletta, Luca Rinaldi, Ferdinando Carlo Sasso, Luigi Elio Adinolfi, Alfredo Caturano

**Affiliations:** 1 Department of Infectious Diseases, Azienda Ospedaliera Regionale San Carlo, 85100 Potenza, Italy; 2 Liver Unit, Azienda Ospedaliera di Rilievo Nazionale S. G. Moscati, “A. Landolfi” Hospital, 83029 Solofra, Italy; riccardo.nevola@unicampania.it; 3 Department of Anesthesiology and Intensive Care, Azienda Ospedaliera Regionale San Carlo, 85100 Potenza, Italy; fannia.barletta@ospedalesancarlo.it; 4 Department of Medicine and Health Science “Vincenzo Tiberio”, University of Molise, 86100 Campobasso, Italy; luca.rinaldi@unimol.it; 5 Department of Advanced Medical and Surgical Sciences, University of Campania “Luigi Vanvitelli”, 80138 Naples, Italy; ferdinandocarlo.sasso@unicampania.it (F.C.S.); luigielio.adinolfi@unicampania.it (L.E.A.); 6 Department of Human Sciences and Promotion of the Quality of Life, San Raffaele Roma Open University, 00166 Rome, Italy; alfredo.caturano@uniroma5.it

**Keywords:** metabolic syndrome, multidrug-resistant infections, antimicrobial resistance, immunometabolism, gut microbiota, insulin resistance, dysbiosis, host–pathogen interaction

## Abstract

Over the past two decades, metabolic syndrome (MetS) and infections caused by multidrug-resistant (MDR) pathogens have emerged as converging global health challenges. Traditionally investigated as separate entities, accumulating evidence increasingly supports a bidirectional relationship between them, mediated by chronic inflammation, immune dysregulation, gut microbiota alterations, and antibiotic-driven expansion of the resistome. This narrative review examines the complex immunometabolic interplay linking MetS and MDR infections, focusing on molecular mechanisms, clinical implications, and prospective research directions. A systematic literature search was conducted using major databases, including PubMed and Scopus, targeting studies from the last 15 years that explore the interface between metabolic dysfunction and antimicrobial resistance. Particular attention is given to key immunometabolic pathways such as the IRS–PI3K–AKT–mTOR axis; the contribution of visceral adiposity and Toll-like receptor (TLR)-mediated inflammation; and the role of gut dysbiosis in augmenting both susceptibility to infections and metabolic derangements. Evidence is presented supporting the hypothesis that MetS increases host vulnerability to MDR pathogens, while chronic MDR infections may reciprocally induce systemic metabolic reprogramming. Viral infections with established metabolic sequelae (e.g., HIV, hepatitis C virus [HCV], and cytomegalovirus [CMV]) are also considered to broaden the conceptual framework. Although current data remain largely associative and fragmented, the emerging MetS–MDR syndemic model poses substantial challenges for translational research, antimicrobial stewardship, and personalized therapeutic strategies. Recognizing this reciprocal relationship is pivotal for refining infection risk stratification, optimizing treatment, and informing public health policies. Further investigations are warranted to elucidate the magnitude and directionality of this association and to identify predictive immunometabolic biomarkers that may guide targeted interventions in high-risk populations.

## 1. Introduction

Over the past two decades, two distinct yet increasingly interrelated global health threats have emerged: metabolic syndrome (MetS) and infections caused by multidrug-resistant (MDR) pathogens. MetS, characterized by a cluster of cardiometabolic abnormalities—including visceral adiposity, insulin resistance, dyslipidemia, and hypertension—affects approximately 25% of the adult global population and significantly increases the risk of type 2 diabetes mellitus (T2DM), cardiovascular disease, and metabolic dysfunction-associated steatotic liver disease (MASLD) [1].

Concurrently, antimicrobial resistance (AMR) has risen sharply worldwide, with MDR infections estimated to have contributed to 4.95 million deaths in 2019, of which 1.27 million were directly attributable to resistant pathogens [2]. MDR organisms such as *Klebsiella pneumoniae*, *Acinetobacter baumannii*, and *Pseudomonas aeruginosa*—key members of the ESKAPE group—display remarkable adaptability, robust biofilm formation, and efficient acquisition of resistance genes via plasmids and integrons, posing ongoing challenges to clinical management and hospital infection control [3].

Although historically regarded as separate epidemics, MetS and MDR infections may interact through complex and underappreciated mechanisms. Emerging data suggest a bidirectional relationship mediated by chronic inflammation, immune dysregulation, alterations of the gut microbiota, and antibiotic-driven expansion of the intestinal resistome [4].

On one hand, the low-grade chronic inflammation and immune impairment characteristic of MetS may increase susceptibility to colonization and persistence of MDR pathogens. Conversely, chronic or recurrent MDR infections may exacerbate immunometabolic stress, contributing to insulin resistance, gut dysbiosis, and systemic metabolic disturbances [5].

Despite these emerging associations, the magnitude and directionality of the relationship between MetS and MDR infections remain insufficiently quantified, with a paucity of longitudinal or meta-analytic data. Moreover, the current literature lacks an integrative conceptual framework encompassing metabolic, immunological, and microbiological dimensions.

The additional contribution of chronic viral infections—such as human immunodeficiency virus (HIV), hepatitis C virus (HCV), and cytomegalovirus (CMV)—which influence both antimicrobial resistance and metabolic dysfunction, further complicates this multifactorial interplay [6,7].

This review aims to provide a translational synthesis of the bidirectional relationship between MetS and MDR infections, emphasizing underlying molecular mechanisms, immunometabolic pathways, and clinical implications. Although the proposed MetS–MDR interaction model remains hypothetical, outlining such a framework may stimulate hypothesis-driven research and novel therapeutic perspectives.

Recognizing the limitations of the current evidence, we adopt a cautious interpretative approach, focusing on associations rather than causality, and propose the MetS–MDR relationship as a potential syndemic model. This perspective encourages re-examination of antimicrobial stewardship, infection prevention strategies, and chronic disease management within an increasingly complex immunometabolic context.

### 1.1. Framing the Problem

The global dissemination of MDR bacterial infections has emerged as a critical public health concern, prompting high-priority action from the World Health Organization (WHO) [8].

Pathogens such as *K. pneumoniae*, *A. baumannii*, and *P. aeruginosa* are notable for their capacity to acquire resistance genes through plasmids, transposons, and integrons, as well as for their pronounced biofilm-forming ability, which facilitates persistence and antimicrobial tolerance [3,9,10].

In parallel, MetS has reached epidemic proportions, affecting approximately one in four adults worldwide [1]. The metabolic disturbances characteristic of MetS are increasingly recognized as risk factors for infection. Specifically, MetS impairs innate immune responses by reducing phagocytic function, compromising natural killer (NK)-cell activity, and skewing macrophage polarization toward pro-inflammatory phenotypes, thereby creating a permissive environment for bacterial colonization [11].

Within this context, *A. baumannii* exhibits remarkable ecological plasticity, colonizing not only deep tissues but also the oral cavity of individuals with metabolic disorders, where dysbiosis, inflammation, and local immunosuppression establish a favorable niche [12].

Metagenomic analyses have revealed the selective enrichment of *A. baumannii* in patients with diabetes, obesity, or liver dysfunction, highlighting the influence of host metabolic status on microbiota composition [13].

*K. pneumoniae* also demonstrates heightened virulence in metabolically compromised hosts, with its biofilm formation and immune evasion mechanisms amplified under hyperglycemic or steatotic conditions [14]. The hypermucoviscous ST11 clone, frequently harboring KPC-type carbapenemases, shows a predilection for immunocompromised individuals and is associated with severe and recurrent infections [15].

Conversely, growing evidence suggests that chronic or relapsing MDR infections may themselves induce or accelerate systemic metabolic dysfunction [16]. Through toll-like receptor activation, sustained inflammatory signaling (e.g., IL-6, TNF-α, CRP), and endotoxin release, these infections may chronically disrupt immunometabolic homeostasis, potentially triggering a MetS-like phenotype in previously healthy individuals [17].

This bidirectional trajectory carries significant clinical implications. In hospitalized patients, the coexistence of MetS and MDR infections correlates with worse prognoses, prolonged hospital stays, increased mortality, and greater therapeutic complexity [18].

Altered pharmacokinetics in individuals with obesity or hepatic steatosis, antibiotic-induced selective pressure on microbial communities, and the metabolic side effects of broad-spectrum antimicrobials underscore the need to reassess infection risk management in metabolically vulnerable patients [19].

### 1.2. Aim and Rationale of the Review

In light of the expanding literature on the MetS–MDR interaction, a comprehensive translational analysis of this relationship is urgently warranted. This review aims to elucidate the reciprocal connections between these two global health challenges, with particular emphasis on molecular, immunological, and clinical mechanisms.

The primary objective is to investigate how the immunometabolic alterations characteristic of MetS establish a permissive environment that facilitates colonization, persistence, and enhanced virulence of MDR pathogens. Infections in metabolically compromised patients frequently exhibit increased severity and diminished therapeutic responsiveness, complicating clinical management [4].

We further examine the emerging concept of a “post-infectious MetS” model, which hypothesizes that chronic or recurrent MDR infections may act as initiators of metabolic remodeling, fostering sustained inflammation, gut microbiota dysbiosis, and insulin resistance. This framework raises critical questions regarding the long-term metabolic sequelae of infection and necessitates further research.

Moreover, the review addresses the pharmacokinetic and pharmacodynamic implications of MetS on antimicrobial therapy. Alterations in drug absorption, distribution, metabolism, and excretion in metabolically impaired individuals may contribute to increased risk of treatment failure, drug toxicity, and selection of resistance [19].

By highlighting these therapeutic challenges, we aim to identify gaps in existing treatment paradigms. The clinical implications of the MetS–MDR interplay will also be discussed, encompassing the need for improved diagnostic modalities, risk stratification frameworks, and optimized strategies for therapeutic monitoring.

Through an integrated synthesis of preclinical and clinical evidence, this review aspires to advance a novel conceptual framework linking host metabolic status and antimicrobial resistance, ultimately promoting the development of targeted, holistic therapeutic interventions.

### 1.3. Materials and Methods

This narrative review integrates molecular, immunological, and clinical evidence concerning the proposed bidirectional relationship between metabolic syndrome (MetS) and infections caused by multidrug-resistant (MDR) pathogens. Although not conducted in accordance with PRISMA guidelines, stringent selection criteria were applied to ensure inclusion of high-quality, relevant literature.

### 1.4. Data Sources and Search Strategy

A comprehensive literature search was performed across PubMed, Scopus, and Web of Science, covering publications from January 2005 to March 2025. The search strategy combined Medical Subject Headings (MeSH) and free-text terms, including the following:

- “metabolic syndrome” or “insulin resistance” or “obesity” or “visceral adiposity”
- and
- “multidrug-resistant bacteria” or “MDR pathogens” or “antimicrobial resistance” or “AMR”
- and
- “inflammation” or “immune dysfunction” or “gut microbiota” or “dysbiosis” or “resistome”

Reference lists of selected studies were manually screened to identify additional relevant publications.

### 1.5. Inclusion and Exclusion Criteria

#### 1.5.1. Inclusion Criteria

- Original research articles, meta-analyses, and systematic or narrative reviews;
- Studies exploring the intersection between metabolic dysfunction and antimicrobial resistance;
- English-language articles published in peer-reviewed journals;
- Human studies, animal models, or in vitro experiments with mechanistic relevance.

#### 1.5.2. Exclusion Criteria

- Case reports, editorials, letters, and conference abstracts;
- Studies unrelated to MetS or MDR infections;
- Non-English articles without accessible translations.

### 1.6. Data Extraction and Synthesis

Thematic synthesis focused on the following domains:

- Shared immunometabolic mechanisms (e.g., chronic inflammation, impaired insulin signaling, gut microbiota disruption);
- Impact of MetS on susceptibility to and outcomes of MDR infections;
- Effects of MDR infections and antimicrobial exposure on metabolic regulation;
- Key molecular pathways (e.g., IRS–PI3K–AKT–mTOR, TLR-mediated signaling);
- Therapeutic implications and candidate targets for intervention.

Figures and tables were developed to illustrate mechanistic interactions and bidirectional relationships, with special attention to *K. pneumoniae*, *A. baumannii*, *P. aeruginosa*, and resistant viral pathogens such as HIV and HBV.

### 1.7. Limitations of the Methodology

As a narrative review, this work does not provide a systematic or quantitative meta-analysis. Selection and publication bias cannot be excluded. Nevertheless, the narrative format facilitates broader conceptual integration and critical appraisal of mechanistically diverse studies.

### 1.8. Methodological Flowchart

**Figure 1:**
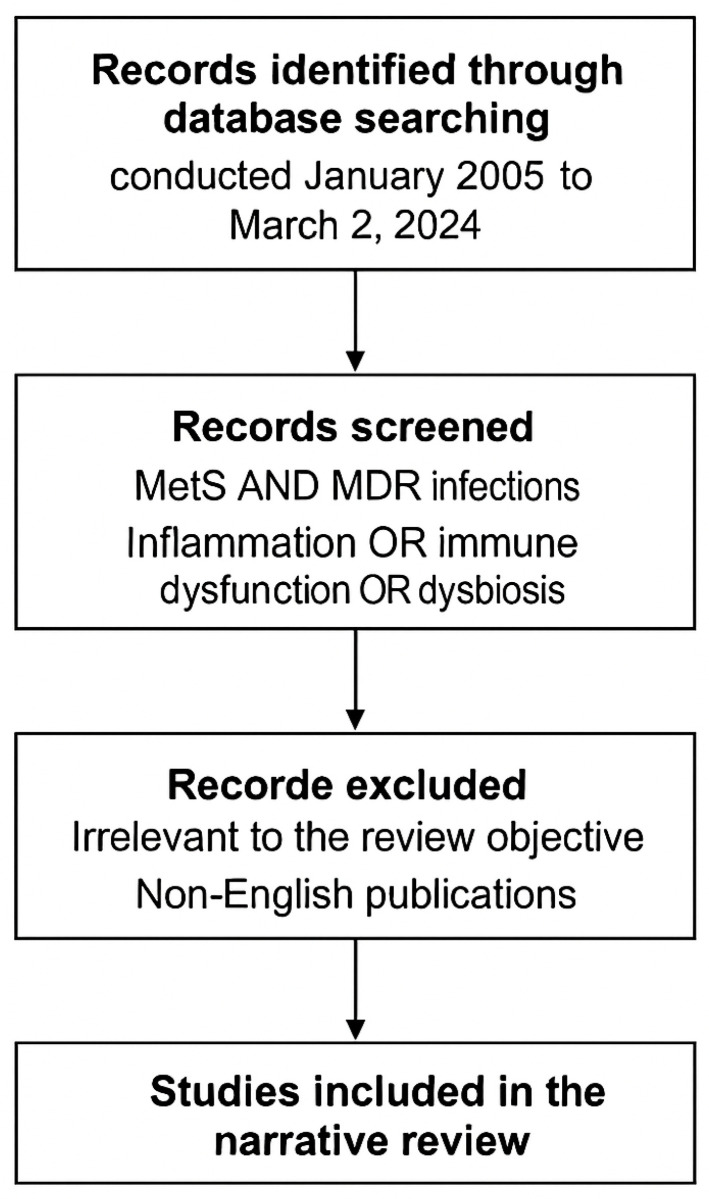
Flow diagram illustrating the methodological steps of the literature selection process used in this narrative review. Records were identified through database searches (PubMed, Scopus, and Web of Science) spanning January 2005 to March 2025, using combined MeSH and free-text terms related to metabolic syndrome, multidrug-resistant (MDR) infections, inflammation, immune dysfunction, and dysbiosis. Studies irrelevant to the review topic or not published in English were excluded. The remaining records were included based on relevance to the MetS–MDR interface, emphasizing mechanistic, clinical, and microbiome-related aspects. Image created using BioRender accessed via web application, version as of April 2025), GraphPad Prism version 10.1.2-2024), and PowerPoint for Microsoft 365, version 2404.

A schematic flow diagram (Figure 1) summarizes the methodological steps of the literature selection process, including database queries, screening criteria, and exclusion parameters.

## 2. Metabolic Syndrome: Epidemiology, Clinical Burden, and Molecular Basis

### 2.1. Definition and Diagnostic Criteria

Metabolic syndrome (MetS), historically referred to as “insulin resistance syndrome” or “syndrome X,” encompasses a cluster of metabolic abnormalities—including hyperglycemia, dyslipidemia, visceral obesity, and arterial hypertension—that collectively confer a markedly increased risk for type 2 diabetes mellitus (T2DM), cardiovascular disease, and metabolic dysfunction-associated steatotic liver disease (MASLD) [20,21].

The contemporary conceptualization of MetS was first formally described by Gerald Reaven in 1988, who identified insulin resistance as the central pathophysiological hallmark [22].

Subsequently, multiple international bodies have proposed diagnostic criteria for MetS, including the World Health Organization (1999) [23], the International Diabetes Federation (2005), the National Cholesterol Education Program—Adult Treatment Panel III (NCEP-ATP III, 2001), and most recently, the Joint Interim Statement (2009), which sought to harmonize preceding definitions [24].

According to this latest consensus, a diagnosis of MetS is established when at least three of the following five criteria are fulfilled: (1) elevated waist circumference based on sex- and ethnicity-specific cutoffs; (2) serum triglycerides ≥ 150 mg/dL; (3) high-density lipoprotein (HDL) cholesterol < 40 mg/dL in men and <50 mg/dL in women; (4) blood pressure ≥ 130/85 mmHg or ongoing antihypertensive treatment; and (5) fasting plasma glucose ≥ 100 mg/dL or current treatment for hyperglycemia [25].

Table 1 summarizes the five diagnostic components delineated by the Joint Interim Statement (2009), which collectively enable the identification of individuals with Metabolic Syndrome.

### 2.2. Global Epidemiology and Emerging Trends

Metabolic syndrome (MetS) has emerged as a critical global health challenge, with its prevalence steadily increasing in both high-income and low-to-middle-income countries [1]. Current estimates suggest that nearly one in four adults worldwide fulfill the diagnostic criteria for MetS [1,26].

In the United States, data from the National Health and Nutrition Examination Survey (NHANES) 2011–2016 report a prevalence of 34.7%, highlighting the substantial burden in high-income settings [27]. Comparable prevalence rates have been documented in South America; for instance, national surveys in Brazil reveal figures ranging from 29% to 38.4%, with notable variability across sex and age groups [28].

Mexico exhibits one of the highest global prevalence rates, with a meta-analysis encompassing over 10,000 adults indicating a prevalence of 41% [29]. In China, prevalence estimates range from 24% to 32%, with marked heterogeneity among ethnic subpopulations, reflecting the complex interplay between genetic predisposition and environmental factors [30].

In Europe, the epidemiological landscape of MetS is similarly heterogeneous. A large multicenter study estimated an average adult prevalence of 24.3%, with comparable rates between males and females [31]. Prevalence notably increases with age, exceeding 30% among individuals aged 70 years and older [26]. Regional differences in the predominant clusters of MetS components have also been observed; for example, the combination of elevated triglycerides, hypertension, and abdominal obesity is more frequently reported in the United Kingdom, Sardinia, and Germany compared to Sweden or Spain [31].

Of particular concern is the rising incidence of MetS among younger populations. Studies investigating obese children and adolescents report prevalence rates ranging from 6% to 39%, contingent upon the diagnostic criteria applied [32,33]. This early onset is especially alarming, as it forecasts an increased future burden of chronic metabolic and cardiovascular diseases [34].

### 2.3. Pathophysiological Basis: Chronic Inflammation, Insulin Resistance, and Immune Dysfunction

Metabolic syndrome (MetS) is a multifactorial disorder characterized by the complex interplay between metabolic abnormalities and immune dysregulation, which perpetuate one another within a self-sustaining pathogenic cycle [35]. Central to its pathophysiology is visceral adipose tissue, now recognized not only as a passive energy reservoir but also as a metabolically active endocrine organ exerting pivotal immunometabolic regulatory functions [36].

#### 2.3.1. Visceral Adipose Tissue as a Pro-Inflammatory Organ

In individuals with MetS, hypertrophy and dysfunction of visceral adipose tissue promote the secretion of a broad spectrum of bioactive mediators, including pro-inflammatory cytokines such as tumor necrosis factor-α (TNF-α) and interleukin-6 (IL-6), adipokines—most notably leptin and adiponectin—and pro-atherogenic molecules [37,38].

These mediators contribute to the establishment of a chronic inflammatory milieu, characterized by persistent immune activation and disruption of metabolic homeostasis [39]. Leptin exerts both pro-inflammatory and pro-atherogenic effects [40], whereas adiponectin—a key anti-inflammatory and insulin-sensitizing adipokine—is typically diminished in circulation, thereby exacerbating metabolic dysfunction [41].

**Figure 2:**
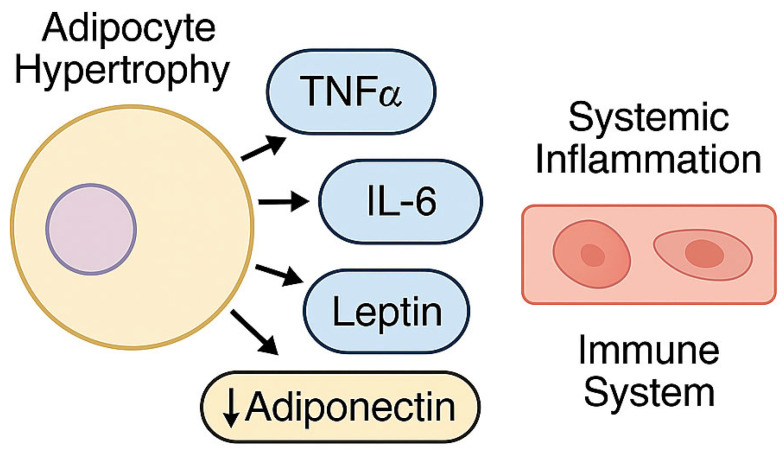
Hypertrophy of adipocytes in the context of metabolic syndrome leads to increased secretion of pro-inflammatory cytokines, including tumor necrosis factor-alpha (TNF-α), interleukin-6 (IL-6), and the adipokine leptin, along with a reduction in adiponectin, an anti-inflammatory and insulin-sensitizing hormone. This imbalance contributes to systemic inflammation and immune system activation. Arrows indicate the directional relationship between adipocyte hypertrophy, altered adipokine secretion, and downstream immunometabolic effects. Image created using BioRender accessed via web application, version as of April 2025), GraphPad Prism version 10.1.2-2024), and PowerPoint for Microsoft 365, version 2404.

The pro-inflammatory cytokine environment and dysregulated adipokine secretion associated with hypertrophic adipose tissue play a pivotal role in sustaining systemic low-grade inflammation and immune activation in the context of MetS (Figure 2). This immunometabolic imbalance may critically modulate host susceptibility to infection and colonization by multidrug-resistant (MDR) pathogens.

#### 2.3.2. Low-Grade Chronic Inflammation and Innate Immune Activation

A hallmark feature of metabolic syndrome (MetS) is the presence of low-grade, systemic inflammation that develops insidiously and persists chronically [35,42]. This persistent inflammatory state is predominantly maintained by activation of innate immune pathways, notably those mediated by toll-like receptors (TLRs). Among these, TLR4 plays a central role in recognizing both exogenous microbial ligands, such as bacterial lipopolysaccharides, and endogenous danger-associated molecular patterns (DAMPs), including saturated fatty acids [17].

Upon ligand binding, TLRs trigger the nuclear factor kappa-light-chain-enhancer of activated B cells (NF-κB) signaling cascade, resulting in transcriptional upregulation of pro-inflammatory cytokines, chemokines, and adhesion molecules [17]. Although this response initially serves protective functions, chronic activation under metabolic stress conditions—such as nutrient excess, lipotoxicity, and cellular injury—renders the pathway maladaptive. Consequently, sustained inflammation ensues, driving progressive tissue dysfunction [43].

#### 2.3.3. Insulin Resistance: A Central Pathophysiological Node in MetS

Insulin resistance constitutes a central pathophysiological mechanism underlying metabolic syndrome (MetS) [22,44]. It is defined by a diminished responsiveness to insulin in peripheral tissues, leading to impaired glucose uptake in skeletal muscle and adipose tissue, increased hepatic gluconeogenesis, and unrestrained lipolysis [45].

These metabolic derangements culminate in hyperglycemia, compensatory hyperinsulinemia, and elevated circulating free fatty acid levels [21,46]. Notably, these alterations not only reflect disease progression but also actively contribute to the amplification of inflammatory signaling pathways. Through this mechanism, they sustain immune activation and further exacerbate insulin resistance, establishing a self-perpetuating pathogenic cycle [46].

#### 2.3.4. Adaptive Immune Dysfunction

Immune dysregulation in metabolic syndrome (MetS) extends beyond the innate immune system to encompass adaptive immunity [4,36]. Current evidence demonstrates a polarization toward pro-inflammatory T helper 1 (Th1) and T helper 17 (Th17) cell subsets, along with increased infiltration of cytotoxic CD8^+^ T lymphocytes within inflamed adipose tissue [36].

Concomitantly, a reduction in regulatory T cells (Tregs), which normally exert immunosuppressive and anti-inflammatory functions, has been documented [36]. This alteration in the balance between effector and regulatory lymphocyte populations intensifies the inflammatory milieu and sustains immune system activation [47].

**Figure 3:**
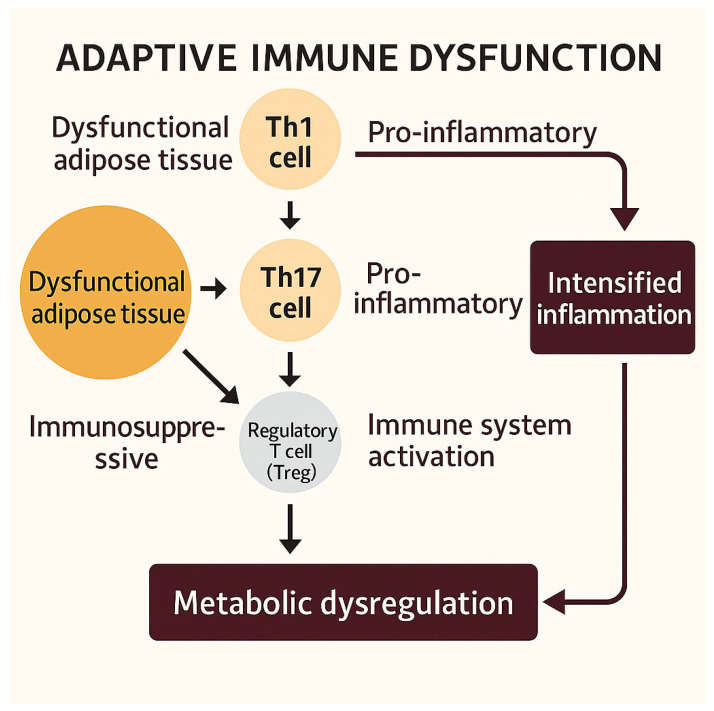
The figure illustrates the contribution of dysfunctional adipose tissue to adaptive immune dysregulation in metabolic syndrome. The expansion and inflammation of visceral fat promote the differentiation of pro-inflammatory Th1 and Th17 T-cell subsets, while concurrently reducing the function and number of immunosuppressive regulatory T cells (Tregs). This immune imbalance leads to intensified inflammation and systemic immune activation, ultimately contributing to metabolic dysregulation. Arrows represent directional relationships between immune cell subsets and pathological outcomes. Colors differentiate cell types (yellow/orange: Th1/Th17; gray: Treg), tissue origin (light orange: adipose tissue), and downstream consequences (dark red: inflammation and metabolic dysregulation). Image created using BioRender accessed via web application, version as of April 2025), GraphPad Prism version 10.1.2-2024), and PowerPoint for Microsoft 365, version 2404.

The predominance of Th1 and Th17 responses, combined with Treg depletion, establishes a pro-inflammatory microenvironment that contributes to the progression of metabolic dysfunction (Figure 3).

#### 2.3.5. Gut Dysbiosis and Metabolic Endotoxemia

Gut dysbiosis is increasingly recognized as a critical contributor to the pathogenesis of metabolic syndrome (MetS) [48]. Individuals affected typically display reduced microbial diversity, an altered Firmicutes-to-Bacteroidetes ratio, and increased intestinal permeability [48].

These perturbations facilitate translocation of microbial endotoxins—particularly lipopolysaccharide (LPS)—from the intestinal lumen into the systemic circulation, resulting in a pro-inflammatory condition termed metabolic endotoxemia [49].

This state acts as a potent stimulus for systemic immune activation, sustaining chronic inflammation and exacerbating insulin resistance. Moreover, the dynamic interplay among dysbiotic microbiota, compromised epithelial barrier integrity, and host immune responses represents a plausible mechanistic nexus linking MetS with increased susceptibility to infection [48,50].

## 3. Multidrug-Resistant Infections: Definitions, Molecular Basis, Epidemiology, and Clinical Impact

### 3.1. Definitions and Classification of Multidrug Resistance

Multidrug-resistant (MDR) pathogens are defined as microorganisms demonstrating non-susceptibility to at least one agent in three or more major antimicrobial categories [51]. This classification further encompasses extensively drug-resistant (XDR) organisms, which remain susceptible to only one or two antimicrobial classes, and pan-drug-resistant (PDR) strains, which exhibit resistance to all currently available antibiotics [52]. These categories highlight the escalating therapeutic impasse imposed by antimicrobial resistance and its profound implications for global health.

Among the most clinically relevant MDR pathogens are members of the ESKAPE group—*Enterococcus faecium*, *Staphylococcus aureus*, *Klebsiella pneumoniae*, *Acinetobacter baumannii*, *Pseudomonas aeruginosa*, and *Enterobacter* spp.—which collectively account for the majority of nosocomial infections and epitomize resistance-driven morbidity and mortality [53,54].

### 3.2. Molecular Mechanisms of Resistance

The emergence of antimicrobial resistance among these pathogens is driven by a convergence of sophisticated molecular mechanisms. These encompass the production of extended-spectrum β-lactamases (ESBLs) and carbapenemases, including KPC, NDM, VIM, and OXA variants; ribosomal target methylation; alterations in penicillin-binding proteins; upregulation of efflux pumps; porin loss or mutation; and enzymatic modification of antibiotic targets [55,56].

Horizontal gene transfer, particularly mediated by plasmids, facilitates the rapid dissemination of resistance determinants both within and across bacterial species, often resulting in the co-localization of multiple resistance genes within a single strain [57]. The interplay of these mechanisms accelerates the evolution of extensively drug-resistant (XDR) and pan-drug-resistant (PDR) phenotypes, thereby progressively undermining the efficacy of conventional antimicrobial therapies [55].

Table 2 summarizes the principal molecular mechanisms underlying resistance in multidrug-resistant pathogens.

### 3.3. Global Epidemiology and Burden of Disease

According to the Global Burden of Disease 2019 study, antimicrobial resistance (AMR) was associated with an estimated 4.95 million deaths globally, of which 1.27 million were directly attributable to infections caused by multidrug-resistant (MDR) pathogens [58]. The burden is particularly severe in low- and middle-income countries (LMICs), where hospital-based resistance to third-generation cephalosporins among Gram-negative bacteria—such as *Escherichia coli* and *Klebsiella pneumoniae*—often exceeds 50% [59,60].

In Europe, surveillance data from the European Centre for Disease Prevention and Control (ECDC) estimate approximately 33,000 annual deaths attributable to MDR infections, accompanied by an economic burden exceeding EUR 900 million per year [61]. Without effective global interventions, AMR is projected to cause up to 10 million deaths annually by 2050, potentially surpassing cancer as the leading cause of mortality worldwide [62].

### 3.4. Clinical Manifestations and Healthcare Impact

Multidrug-resistant (MDR) pathogens are implicated in a broad spectrum of clinical presentations, ranging from persistent colonization to fulminant, life-threatening infections. *Klebsiella pneumoniae*, particularly strains producing KPC-type carbapenemases (KPC-Kp), represents a major etiologic agent of severe nosocomial infections—including bloodstream infections and ventilator-associated pneumonia—with hospital mortality rates exceeding 40% in several patient cohorts [55,63].

*Acinetobacter baumannii* is notably prevalent in intensive care units, where its ability to form biofilms, resist desiccation, and withstand multiple antibiotic classes renders it a formidable therapeutic challenge. Extensively drug-resistant (XDR) strains of *A. baumannii* have been associated with mortality rates exceeding 50%, particularly among mechanically ventilated patients and those with sepsis [9,29].

*Pseudomonas aeruginosa*, frequently isolated in immunocompromised hosts, individuals with cystic fibrosis, or patients with indwelling medical devices, combines intrinsic resistance with the capacity to acquire additional potent resistance mechanisms, including metallo-β-lactamases such as VIM and IMP. Carbapenem-resistant *P. aeruginosa* is associated with prolonged hospital stays and increased risk of therapeutic failure [10,64,65].

*Staphylococcus aureus*, particularly methicillin-resistant strains (MRSA), remains a globally prevalent pathogen responsible for severe infections including bacteremia, endocarditis, osteomyelitis, and pneumonia, with case-fatality rates reaching 20–30% in high-risk populations [66,67]. The emergence of community-associated MRSA (CA-MRSA) has further complicated clinical management due to enhanced virulence and transmission dynamics [68].

*Enterococcus faecium*, especially in its vancomycin-resistant form (VRE), poses significant risks in immunocompromised and transplant patients [69]. The vanA gene cluster confers high-level resistance to both vancomycin and teicoplanin, necessitating treatment with alternative agents such as linezolid or daptomycin, which are costly and associated with notable toxicity [70,71].

Beyond clinical outcomes, the economic impact of MDR outbreaks is considerable. For instance, a 2015 outbreak of NDM-1-producing *K. pneumoniae* in the Netherlands involving 29 patients incurred total costs of USD 804,263, largely attributable to lost hospital revenue and containment measures [72].

At a systemic level, antimicrobial resistance constitutes an existential threat to modern healthcare systems. Without decisive interventions, cumulative global economic losses are projected to reach USD 100 trillion by 2050 [62].

Table 3 summarizes the clinical severity and economic burden associated with principal MDR pathogens.

## 4. Pathophysiological Intersections Between Metabolic Syndrome and Antimicrobial Resistance

The interplay between metabolic syndrome (MetS) and infections caused by multidrug-resistant (MDR) organisms is increasingly recognized as a complex immunometabolic phenomenon [50]. Several convergent mechanisms—including chronic inflammation, immune dysregulation, insulin resistance, and alterations in gut microbiota—create a biological milieu that simultaneously promotes metabolic dysfunction and facilitates the expansion of antimicrobial resistance [73].

Although direct causality remains to be definitively established, a growing body of mechanistic evidence supports the conceptualization of a syndemic model, wherein metabolic and infectious processes mutually reinforce each other through reciprocal biological amplification [73].

### 4.1. Chronic Inflammation and Immune Dysregulation

Low-grade chronic inflammation is a defining feature of metabolic syndrome (MetS) [35]. Visceral adipose tissue—particularly when expanded and dysfunctional—serves as a persistent source of pro-inflammatory cytokines, including tumor necrosis factor-α (TNF-α), interleukin-6 (IL-6), interleukin-1β (IL-1β), and monocyte chemoattractant protein-1 (MCP-1), as well as reactive oxygen species [39].

This pro-inflammatory milieu drives a shift in both innate and adaptive immune responses: macrophages polarize toward the M1 pro-inflammatory phenotype, while T cells preferentially differentiate into Th1 and Th17 subsets, accompanied by a reduction in the number and suppressive function of regulatory T cells (Tregs) [36]. This dysregulated immune profile impairs multiple arms of host defense, including phagocytic capacity, type I interferon production, antigen presentation, and natural killer (NK) cell function [4].

As a result, host susceptibility to colonization and persistence of MDR pathogens such as *Klebsiella pneumoniae* and *Acinetobacter baumannii* is enhanced, as these organisms exploit compromised immune surveillance to establish infection [36].

Table 4 delineates the core immunometabolic mechanisms underlying the heightened vulnerability of individuals with MetS to multidrug-resistant infections.

### 4.2. Gut Dysbiosis and Expansion of the Resistome

Individuals with metabolic syndrome (MetS) frequently display profound alterations in gut microbiota composition, including reduced microbial diversity, an elevated Firmicutes-to-Bacteroidetes ratio, and the enrichment of pro-inflammatory pathobionts such as *Collinsella*, *Prevotella copri*, and *Bacteroides vulgatus* [50]. This dysbiotic configuration compromises mucosal barrier integrity, leading to increased intestinal permeability and metabolic endotoxemia—a condition marked by the systemic translocation of bacterial lipopolysaccharides (LPS)—which perpetuates inflammation and exacerbates metabolic dysfunction [50].

Simultaneously, the altered gut microbiota functions as a reservoir for antibiotic resistance genes—collectively referred to as the resistome—whose expansion is influenced not only by prior antibiotic exposure but also by intrinsic microbiome shifts associated with obesity and insulin resistance [74]. The loss of beneficial short-chain fatty acid-producing taxa, such as *Faecalibacterium prausnitzii*, further impairs gut barrier function, facilitating the translocation of multidrug-resistant (MDR) *Enterobacteriaceae* [4].

**Figure 4:**
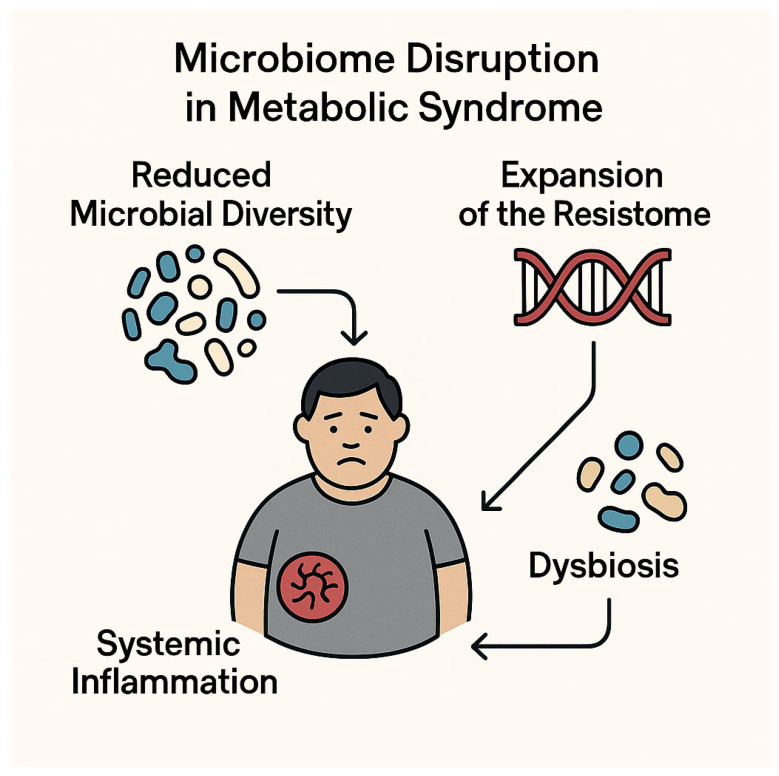
The figure illustrates the key alterations in gut microbiota associated with metabolic syndrome (MetS), including reduced microbial diversity, dysbiosis, and expansion of the intestinal resistome. These changes collectively promote systemic inflammation and contribute to the immunometabolic dysfunction that characterizes MetS. The expansion of the resistome may also facilitate colonization by multidrug-resistant organisms. The interplay between gut dysbiosis and inflammation represents a critical link between host metabolism and antimicrobial resistance. Arrows indicate directional relationships between microbiota alterations and systemic consequences, while colors are used illustratively to distinguish microbial components, resistome expansion, and inflammatory effects. Image created using BioRender accessed via web application, version as of April 2025), GraphPad Prism version 10.1.2-2024), and PowerPoint for Microsoft 365, version 2404.

These microbiota-driven disturbances—including diminished microbial diversity, compositional dysbiosis, and resistome amplification—are now increasingly recognized as central contributors to both the pathogenesis of MetS and its intersection with antimicrobial resistance. By sustaining systemic inflammation and weakening host immune defenses, they enhance susceptibility to MDR colonization and infection (Figure 4).

### 4.3. Insulin Resistance and Host Defense Impairment

Insulin resistance, the central metabolic abnormality in metabolic syndrome (MetS), compromises host immune responses by disrupting key intracellular signaling pathways [46]. Chronic hyperinsulinemia induces serine phosphorylation of insulin receptor substrate-1 (IRS-1), thereby inhibiting the downstream activation of the PI3K–AKT–mTOR axis, a pivotal signaling cascade involved in immune cell metabolism, proliferation, and survival [46].

This impairment hinders T-cell expansion and antigen-specific activation, reduces the phagocytic capacity of macrophages, and interferes with innate immune functions [36]. Moreover, insulin resistance impairs neutrophil chemotaxis and oxidative burst, diminishes natural killer (NK) cell cytotoxicity, and disrupts lipid metabolism [4].

Crucially, the accumulation of free fatty acids and the remodeling of cellular membranes into cholesterol- and sphingolipid-rich lipid rafts may facilitate the intracellular entry and replication of pathogens [75]. Surfactant protein D (SP-D), a key component of respiratory antiviral defense, is functionally impaired in hyperglycemic environments, further increasing susceptibility to viral infections [4].

Despite the strong biological plausibility of these mechanisms, no studies to date have quantitatively assessed whether dysregulation of the PI3K–AKT–mTOR pathway predicts the severity or persistence of MDR infections, highlighting a significant gap in translational research [46].

Table 5 outlines the principal genes, proteins, and molecular pathways mediating the immunometabolic disturbances that contribute to heightened vulnerability to MDR infections, including disruptions in the IRS–PI3K–AKT–mTOR axis and chronic activation of TLR4–NF-κB signaling.

### 4.4. Microbiota–Immune Axis and Resistance Transmission

Preclinical studies have shown that fecal microbiota transplantation (FMT) from individuals with MetS into germ-free or microbiota-depleted mice induces hallmark features of metabolic dysfunction—including impaired glucose tolerance and systemic inflammation—and increases susceptibility to MDR infection with *Klebsiella pneumoniae* [76]. These findings suggest that the gut microbiota may function not only as a reservoir of resistance genes but also as a vector for transmissible traits that amplify vulnerability to both metabolic and infectious diseases.

**Figure 5:**
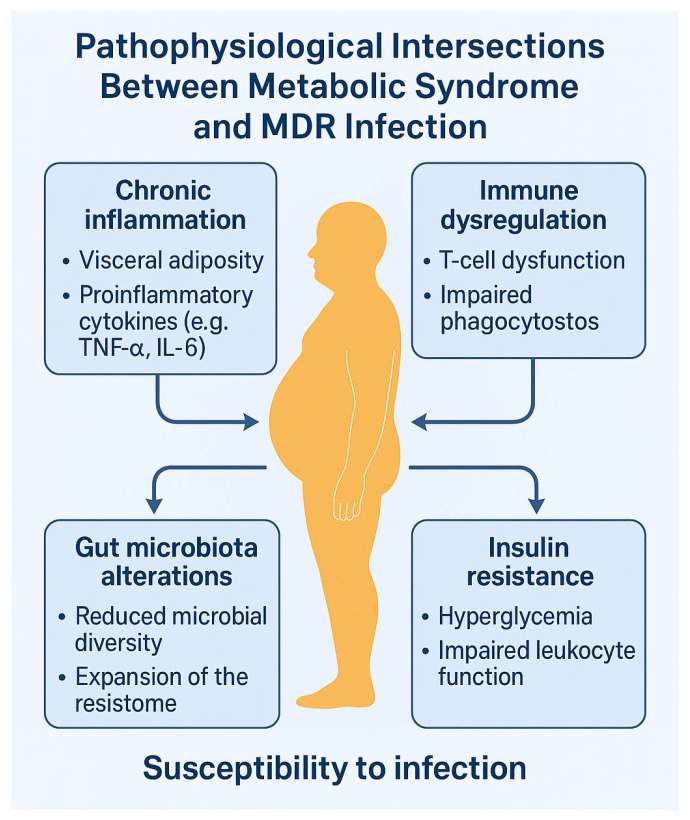
Pathophysiological links between metabolic syndrome and increased susceptibility to multidrug-resistant (MDR) infections. This figure illustrates the key pathophysiological mechanisms through which metabolic syndrome contributes to increased vulnerability to MDR infections. Chronic inflammation driven by visceral adiposity and proinflammatory cytokines (e.g., TNF-α, IL-6), immune dysregulation characterized by T-cell dysfunction and impaired phagocytosis, insulin resistance leading to hyperglycemia and compromised leukocyte function, and gut microbiota alterations with reduced diversity and expansion of the resistome collectively impair host defense mechanisms, ultimately heightening susceptibility to infection. Arrows represent directional relationships among the different pathophysiological components. Colors are used illustratively to distinguish between functional domains and physiological processes. Image created using BioRender accessed via web application, version as of April 2025), GraphPad Prism version 10.1.2-2024), and PowerPoint for Microsoft 365, version 2404.

Chronic activation of pattern recognition receptors, particularly toll-like receptor 4 (TLR4), by bacterial lipopolysaccharide (LPS) and free fatty acids perpetuates a state of metaflammation and immune exhaustion [17]. This feed-forward loop further weakens host defenses against MDR pathogens [77]. These complex interactions are illustrated in Figure 5.

However, the extent to which these findings are applicable to human disease remains unclear. Longitudinal clinical studies investigating microbiota-mediated resistome transmission in metabolically compromised populations are currently lacking, underscoring the need for additional translational and clinical research.

## 5. Impact of Metabolic Syndrome on MDR Infection Risk and Outcomes

Metabolic syndrome (MetS) is increasingly recognized as a key determinant of adverse outcomes in infections caused by multidrug-resistant (MDR) pathogens [73]. This association is driven by a complex array of immunometabolic disturbances—including systemic low-grade inflammation, insulin resistance, gut dysbiosis, and dysregulation of both innate and adaptive immunity—that collectively impair host defense mechanisms and therapeutic responsiveness [35].

Core features of MetS, such as visceral adiposity and poorly controlled type 2 diabetes mellitus (T2DM), compromise antimicrobial immunity and significantly influence infection susceptibility, treatment efficacy, and long-term prognosis [20].

### 5.1. Increased Susceptibility and Delayed Infection Resolution

Individuals with MetS demonstrate heightened vulnerability to infections caused by MDR pathogens—including *Klebsiella pneumoniae* and *Acinetobacter baumannii*—particularly in nosocomial and high-risk healthcare environments [78].

This increased susceptibility arises from multiple converging mechanisms. Chronic metaflammation associated with visceral adiposity fosters a pro-inflammatory cytokine milieu dominated by TNF-α and IL-6. Concurrently, insulin resistance and hyperglycemia impair the function of neutrophils and macrophages, inhibit phagocytosis, and suppress type-I interferon responses [36]. Natural killer (NK)-cell cytotoxicity is reduced, and an imbalance between Th17 and regulatory T cells (Tregs) promotes a state of functional immunoparalysis [39].

These immune alterations hinder effective pathogen clearance and promote prolonged colonization, especially in the gastrointestinal and respiratory tracts [4]. Notably, infections with biofilm-forming MDR organisms are particularly severe and refractory in this context, frequently characterized by delayed resolution, a high microbial burden, and increased recurrence, especially in intensive care and post-surgical settings [55].

### 5.2. Influence on Hospitalization, ICU Stay, and Mortality

MetS has consistently been associated with worse clinical outcomes in patients with severe infections, including those caused by MDR bacteria [79]. During the 2009 H1N1 influenza pandemic, T2DM was shown to triple the risk of hospitalization and quadruple the likelihood of ICU admission [80], a trend subsequently corroborated in bacterial sepsis and nosocomial pneumonia [81].

In patients infected with carbapenemase-producing *K. pneumoniae* (KPC-Kp), obesity (BMI >30) independently predicted higher in-hospital mortality and empirical treatment failure, even after adjustment for comorbidities and illness severity [82]. Similar associations have been observed in critically ill patients with MDR *A. baumannii* infections, where obesity correlates with a prolonged ICU stay, greater use of mechanical ventilation and renal replacement therapy, and increased mortality [83,84].

These clinical outcomes reflect the underlying immunometabolic dysfunction characteristic of MetS, which amplifies infection severity and complicates therapeutic management [85]. Postoperative infectious complications—particularly surgical site infections caused by MDR *Enterobacteriaceae* and carbapenem-resistant *P. aeruginosa*—are also more frequent in patients with T2DM or MASLD [86].

These findings underscore the prognostic relevance of integrating metabolic status into clinical risk stratification, particularly in surgical, oncological, and critical-care settings.

Table 6 presents real-world clinical evidence highlighting the prognostic impact of metabolic syndrome on outcomes in patients with multidrug-resistant infections.

### 5.3. Evidence from Clinical Cohorts and Real-World Studies

Findings from observational cohorts and real-world studies further reinforce the negative prognostic impact of metabolic syndrome (MetS) in the setting of multidrug-resistant (MDR) infections [87]. In a large multicenter study conducted in Spain, diabetes was independently associated with higher 30-day mortality and increased recurrence of MDR bacteremia, irrespective of the appropriateness of the initial antimicrobial therapy [88].

Retrospective analyses have demonstrated that elevated body mass index (BMI) adversely affects infection control and treatment outcomes in severe infections. Specifically, obesity has been linked to increased mortality in ICU patients with sepsis or septic shock [89], as well as to higher in-hospital mortality and empirical treatment failure in individuals with carbapenem-resistant *K. pneumoniae* bacteremia [82].

These associations may be partly explained by pharmacokinetic alterations associated with obesity and metabolic dysfunction—including expanded volume of distribution, reduced tissue penetration, and impaired hepatic metabolism—particularly in the context of metabolic dysfunction-associated steatotic liver disease (MASLD) [90,91]. Moreover, deep-seated infections, which are more frequent in obese patients, further complicate therapeutic success.

Additionally, metabolic dysregulation often leads to prolonged or suboptimal use of broad-spectrum antibiotics, thereby promoting the selection of MDR organisms and exacerbating disruption of the gut microbiota [92].

From a health economics perspective, cost-modeling studies estimate that the coexistence of MetS and MDR infections increases healthcare expenditures by up to 35%, primarily due to prolonged hospital stays, elevated complication rates, and the necessity for expensive antimicrobial regimens [72].

Despite the robustness of these associations, current evidence is predominantly observational and lacks integration across metabolic, microbiological, and clinical domains. There is a pressing need for prospective, longitudinal studies that incorporate metabolic indices—such as HOMA-IR, BMI, and hepatic steatosis grade—into outcome analyses. Such data are essential for identifying predictive markers of infection risk and therapeutic failure, as well as for guiding the development of precision antimicrobial stewardship strategies tailored to metabolically vulnerable populations [93].

**Figure 6:**
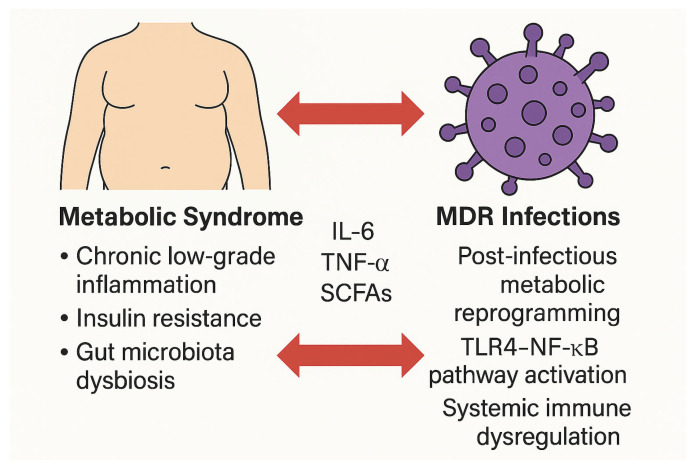
Schematic representation of the bidirectional relationship between metabolic syndrome and multidrug-resistant (MDR) infections. Metabolic syndrome contributes to increased susceptibility to MDR pathogens through chronic low-grade inflammation, insulin resistance, and gut microbiota dysbiosis. Conversely, MDR infections may induce post-infectious metabolic reprogramming and systemic immune dysregulation, primarily via TLR4–NF-κB pathway activation. Key mediators of this interplay include proinflammatory cytokines such as interleukin-6 (IL-6), tumor necrosis factor-α (TNF-α), and alterations in short-chain fatty acids (SCFAs). Arrows indicate the directionality of the interactions between metabolic and infectious processes. Colors are used illustratively to distinguish metabolic alterations, immunological pathways, and microbial factors. Image created using BioRender accessed via web application, version as of April 2025), GraphPad Prism version 10.1.2-2024), and PowerPoint for Microsoft 365, version 2404.

Figure 6 provides a visual summary of the bidirectional interactions described, illustrating how metabolic syndrome enhances susceptibility to MDR infections, while MDR pathogens may reciprocally contribute to immunometabolic dysfunction (Figure 6).

## 6. Can Multidrug-Resistant Infections Contribute to Metabolic Dysfunction?

While metabolic syndrome (MetS) is widely acknowledged as a predisposing factor for infection, an increasing body of evidence suggests a potential reverse pathophysiological trajectory: severe or recurrent infections—particularly those caused by multidrug-resistant (MDR) pathogens—may themselves induce or exacerbate metabolic dysfunction [94].

This emerging concept, situated at the crossroads of immunometabolism and microbiome science, calls for a syndemic and longitudinal reappraisal of infectious sequelae, especially in vulnerable individuals [4,48].

### 6.1. Post-Infectious Inflammation and Metabolic Reprogramming

Persistent immune activation following clinical resolution of MDR infections—particularly when complicated by sepsis or pneumonia—can drive sustained post-infectious metabolic remodeling, with effects lasting weeks to months [16].

Documented metabolic alterations include de novo or persistent hyperglycemia after hospital discharge, mitochondrial dysfunction with impaired oxidative phosphorylation, hypothalamic–pituitary–adrenal (HPA) axis dysregulation with cortisol resistance, and increased insulin resistance mediated by pro-inflammatory cytokines, such as IL-6 and TNF-α [16,43].

At the molecular level, murine models have demonstrated that chronic toll-like receptor (TLR) activation—particularly TLR4—by bacterial endotoxins induces aberrant transcriptional programs in metabolism-related genes, directly impacting hepatic and adipose tissues even in the absence of dietary or genetic predisposition [95]. In these models, hepatic insulin sensitivity remains impaired long after infection resolution, suggesting that metabolic consequences may extend well beyond the acute infectious episode.

This phenomenon is also observed in chronic viral infections associated with drug resistance. In HIV-positive individuals experiencing virologic failure on antiretroviral therapy (ART), mitochondrial dysfunction, visceral adiposity, insulin resistance, and atherogenic dyslipidemia have been reported, attributable to both persistent viral replication and cumulative ART-related toxicity [96].

Similarly, hepatitis B virus (HBV) infection with tenofovir- or lamivudine-resistant mutations is associated with an increased risk of MASLD, likely mediated by nuclear receptor dysregulation (e.g., FXR and PPAR-α), oxidative stress, and impaired intrahepatic lipid metabolism [97].

Collectively, these findings support the hypothesis of a “post-infectious metabolic syndrome,” in which sustained inflammatory and immunometabolic signaling following MDR infections drives long-lasting endocrine and metabolic dysfunction [16].

Nevertheless, the causality and reversibility of these alterations remain poorly understood. Prospective longitudinal studies and interventional trials are urgently needed to determine whether this represents a distinct clinical entity or a transient stress-related adaptation.

**Figure 7:**
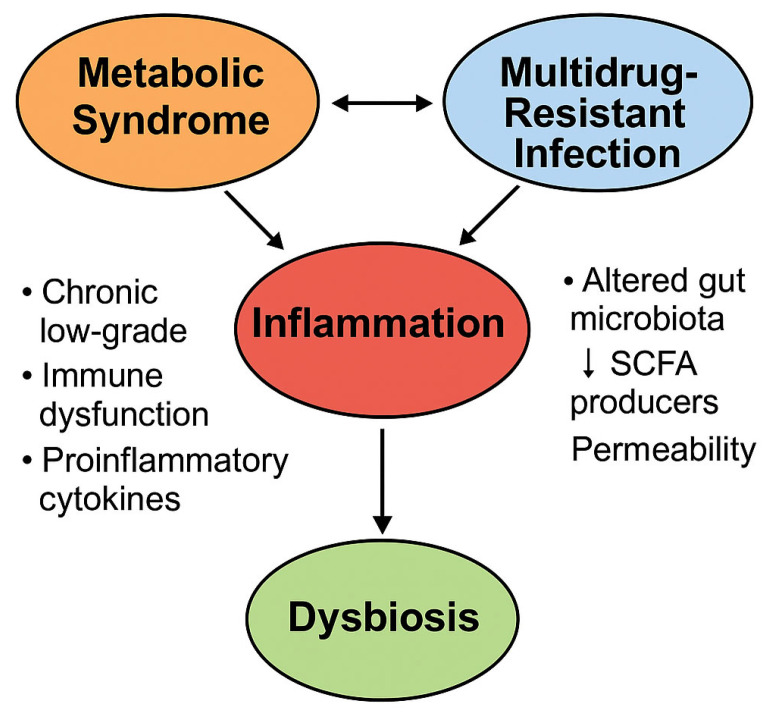
Conceptual diagram illustrating inflammation as a central mediator linking metabolic syndrome and multidrug-resistant (MDR) infections. Chronic low-grade inflammation and immune dysfunction in metabolic syndrome promote proinflammatory cytokine release, while MDR infections exacerbate this process through altered gut microbiota composition, reduced short-chain fatty acid (SCFA) producers, and increased intestinal permeability. This inflammation-driven cascade contributes to progressive dysbiosis and systemic metabolic disruption. Arrows represent directional and reciprocal interactions among metabolic, immune, and microbial factors. Colors are used illustratively to differentiate the domains of host metabolism, immune signaling, and microbiota-derived influences. Image created using BioRender accessed via web application, version as of April 2025), GraphPad Prism version 10.1.2-2024), and PowerPoint for Microsoft 365, version 2404.

As illustrated in Figure 7, inflammation serves as a pivotal mechanistic link between metabolic syndrome and MDR infections, perpetuating a feed-forward loop characterized by gut dysbiosis and immune dysregulation (Figure 7).

### 6.2. Antibiotic-Induced Dysbiosis and the Concept of “Infection-Induced MetS”

A second major mechanism contributing to post-infectious metabolic deterioration is antibiotic-induced intestinal dysbiosis, particularly in patients subjected to prolonged or repeated courses of the broad-spectrum antibiotics commonly employed in the treatment of MDR infections [98].

Agents such as carbapenems, colistin, fourth-generation cephalosporins, and tigecycline profoundly deplete gut microbial diversity, thereby facilitating the selection of resistant strains and promoting the expansion of the intestinal resistome [99]. The concomitant loss of short-chain fatty acid (SCFA)-producing bacteria, including *Faecalibacterium prausnitzii*, increases intestinal permeability (“leaky gut”), promotes systemic bacterial translocation, and activates innate immune responses [48,100].

These alterations sustain a chronic, low-grade inflammatory state with diabetogenic effects, even in the absence of dietary changes [50,101].

Further support for the concept of “infection-induced MetS” arises from preclinical studies utilizing fecal microbiota transplantation (FMT), which demonstrate that microbiota derived from individuals with MetS or chronic MDR infections can induce metabolic phenotypes in germ-free mice, including impaired glucose tolerance, dyslipidemia, and heightened infection susceptibility [102].

Moreover, persistent intestinal colonization with antibiotic-selected Gram-negative MDR organisms—such as KPC-producing *Klebsiella pneumoniae* or ESBL-producing *Escherichia coli*—may perpetuate subclinical inflammation and drive the progression of latent metabolic dysfunction [98].

This phenomenon, increasingly conceptualized as “microbial metabolic imprinting,” underscores the intricate interplay between the intestinal resistome and the host’s metabolism, representing a novel frontier in translational research [103].

Table 7 outlines the bidirectional pathogenic interactions between metabolic syndrome and multidrug-resistant infections, emphasizing the mutual amplification of immune and metabolic disturbances.

## 7. Therapeutic Challenges and Clinical Implications

The bidirectional interplay between metabolic syndrome (MetS) and infections caused by multidrug-resistant (MDR) pathogens introduces a complex set of therapeutic challenges. These include altered pharmacokinetics, immune dysregulation, and microbiome disruption, all of which contribute to reduced antibiotic efficacy, an increased risk of therapeutic failure and toxicity, and enhanced selection of resistant organisms [91,92,104].

To effectively address these challenges, a precision-based therapeutic approach is warranted, one that incorporates individualized pharmacokinetic dosing, robust antimicrobial stewardship, and interventions targeting microbiota integrity and function [105].

### 7.1. Pharmacokinetics and Pharmacodynamics in Metabolic Syndrome

MetS significantly impacts the absorption, distribution, metabolism, and excretion (ADME) of antimicrobial agents. Key components of the syndrome—such as visceral adiposity, insulin resistance, and metabolic dysfunction-associated steatotic liver disease (MASLD)—contribute to an expanded volume of distribution, altered hepatic biotransformation, and variable renal clearance. These pharmacokinetic changes are modulated by both the physicochemical properties of the antimicrobial agent and the metabolic phenotype of the individual patient [106].

Specifically, the combined effects of increased adipose mass, hepatic steatosis, and renal dysfunction variably influence the pharmacokinetic profiles of hydrophilic and lipophilic antimicrobials. Hydrophilic agents may exhibit enhanced distribution and clearance, risking subtherapeutic exposure, while lipophilic drugs may accumulate excessively, increasing toxicity potential.

**Figure 8:**
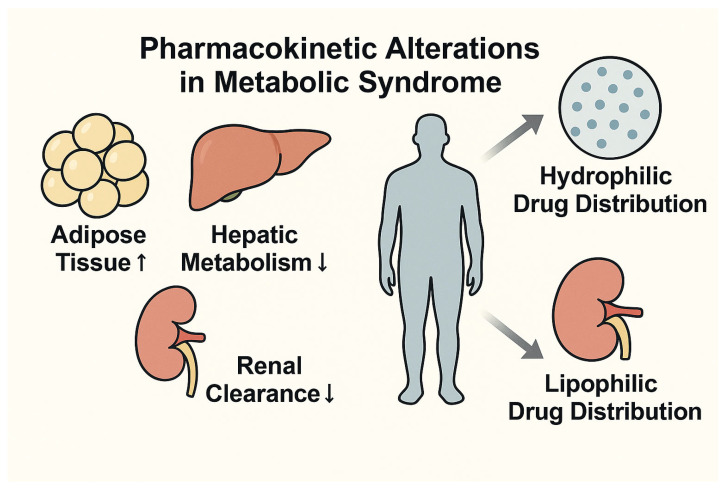
Pharmacokinetic alterations in individuals with metabolic syndrome significantly influence drug disposition and therapeutic outcomes. This schematic illustrates the key pathophysiological changes that affect drug pharmacokinetics in the context of metabolic syndrome: increased adipose tissue, decreased hepatic metabolism, and reduced renal clearance. These alterations modify the distribution profiles of both hydrophilic and lipophilic drugs, with potential implications for dosing, efficacy, and toxicity. The image underscores the need for personalized pharmacotherapy in metabolically compromised patients. Downward arrows (↓) indicate decreased hepatic metabolism and renal clearance. Image created using BioRender accessed via web application, version as of April 2025), GraphPad Prism version 10.1.2-2024), and PowerPoint for Microsoft 365, version 2404.

Visual representation of these pharmacokinetic alterations (Figure 8) may enhance clinical understanding and support the rationale for tailored dosing strategies in patients with MetS, especially in the context of critical care, where metabolic derangements are particularly pronounced.

Hydrophilic antibiotics—such as β-lactams and aminoglycosides—often exhibit increased volumes of distribution and accelerated clearance in patients with MetS, thereby risking subtherapeutic exposure if dosing is not appropriately adjusted based on actual or adjusted body weight [104,107].

In contrast, lipophilic agents—including fluoroquinolones and macrolides—are significantly influenced by increased adipose tissue stores and altered hepatic metabolism via cytochrome P450 pathways, particularly in the setting of hepatic steatosis and inflammation-driven epigenetic dysregulation [104,107,108]. For instance, colistin—characterized by concentration-dependent activity and limited tissue penetration—requires individualized dosing based on ideal or adjusted body weight to optimize therapeutic efficacy while minimizing toxicity [109]. Similarly, aminoglycosides necessitate precise weight-based dose adjustments in obese patients to reduce the risk of nephrotoxicity and ototoxicity [110].

A 2023 systematic review and meta-analysis of randomized controlled trials confirmed the clinical utility of therapeutic drug monitoring (TDM) in critically ill patients, emphasizing the relevance of pharmacokinetic variability and the necessity for individualized antibiotic dosing [111].

Although definitive guidelines from the Infectious Diseases Society of America (IDSA) and the European Society of Clinical Microbiology and Infectious Diseases (ECCMID) are not yet available, expert consensus recommends using total body weight for dosing lipophilic agents, ideal or adjusted body weight for hydrophilic agents, and incorporating hepatic function assessments when prescribing antibiotics metabolized via CYP450 enzymes [104,108].

TDM is strongly recommended for antimicrobials with narrow therapeutic indices—such as vancomycin, aminoglycosides, and β-lactams—especially in intensive care unit (ICU) settings, where pharmacokinetic fluctuations are particularly pronounced [111].

Table 8 outlines the key pharmacokinetic and pharmacodynamic challenges introduced by metabolic syndrome and their implications for antibiotic therapy.

### 7.2. Antimicrobial Stewardship and Optimization of Therapy

The presence of metabolic syndrome (MetS) complicates the implementation of antimicrobial stewardship (AMS) principles. Patients with metabolic comorbidities are frequently perceived as clinically fragile or chronically colonized, resulting in a higher likelihood of receiving empirical broad-spectrum antibiotics [112]. This approach increases antimicrobial selection pressure, exacerbates gut dysbiosis, and accelerates the emergence of resistance [56].

Observational studies in both medical and surgical populations have demonstrated that early antimicrobial de-escalation, guided by microbiological data and shortened treatment durations, can significantly reduce the infection burden and limit the spread of MDR pathogens [113].

Metabolic comorbidities further complicate therapy through altered drug metabolism, increased risk of toxicity, and complex drug–disease interactions [106]. As such, AMS strategies should incorporate metabolic risk stratification, BMI-adjusted dosing regimens, and, where feasible, the preferential use of microbiota-sparing agents in patients with documented dysbiosis or MASLD [104].

Effective stewardship requires multidisciplinary coordination among infectious disease specialists, internists, endocrinologists, and clinical pharmacologists to tailor antimicrobial therapy to the patient’s metabolic profile [112].

Real-world evidence increasingly supports the utility of metabolism-informed stewardship protocols, which enhance treatment precision and contribute to reductions in MDR colonization rates [59,74].

### 7.3. Microbiome-Targeted and Immunometabolic Approaches

Given the pivotal role of gut dysbiosis in both metabolic dysfunction and MDR colonization, microbiota-targeted interventions represent a promising therapeutic avenue [92]. Patients with MetS often harbor a pre-existing imbalance in gut microbial composition, which is further aggravated by repeated antibiotic exposure [48,101]. The result is diminished microbial diversity, expansion of the resistome, and chronic systemic inflammation [74,101].

Interventions such as probiotic and prebiotic supplementation, as well as fecal microbiota transplantation (FMT), have shown efficacy in restoring eubiosis and improving glycemic control, insulin sensitivity, and inflammatory parameters in individuals with MetS or type 2 diabetes mellitus (T2DM) [93].

Additional therapeutic strategies—including polyphenol-based formulations and short-chain fatty acid (SCFA) modulation—are under investigation for their potential to attenuate metaflammation and enhance mucosal immunity [114].

Emerging experimental approaches, such as engineered microbial consortia and immune checkpoint modulators, aim to recalibrate host immune responses in metabolically compromised individuals. These interventions define a novel frontier at the intersection of infectious disease management and precision metabolic medicine [4,114], as summarized in Table 9.

## 8. Health Systems, Surveillance, and Prevention Strategies

The growing convergence between metabolic syndrome (MetS) and infections caused by multidrug-resistant (MDR) pathogens necessitates a fundamental reappraisal of existing prevention, surveillance, and risk management frameworks, both in hospital settings and in communities [5,59].

Public health strategies must incorporate metabolic vulnerability into infectious risk assessment, promoting predictive and personalized approaches aimed at anticipating colonization, limiting transmission, and improving clinical outcomes [115].

### 8.1. Risk Stratification and Screening Protocols in Patients with MetS

Accumulating evidence supporting the bidirectional relationship between MetS and MDR infections highlights the need to incorporate metabolic indicators into infection risk stratification models [5,79]. Patients with visceral obesity, type 2 diabetes mellitus (T2DM), or hepatic steatosis show significantly elevated rates of mucosal colonization by carbapenemase-producing *Enterobacteriaceae* (CRE), extended-spectrum β-lactamase (ESBL)-producing strains, *Klebsiella pneumoniae*, *Acinetobacter baumannii*, and MDR *Escherichia coli* [9,14,79].

These individuals are also characterized by prolonged carriage, increased risk of secondary bloodstream infections, and impaired mucosal immunity due to chronic systemic inflammation [5,11]. In high-intensity care environments such as ICUs, surgical units, and long-term care wards, targeted microbiological surveillance protocols are warranted for patients with metabolic dysfunction [116,117].

Recommended strategies include rectal screening for CRE or ESBL colonization at admission using molecular or selective culture methods and the integration of metabolic parameters—such as BMI, fasting glucose, HOMA-IR, and steatosis grade—into electronic health records (EHRs) to support early risk stratification [118].

### 8.2. Metabolism-Adapted Antimicrobial Stewardship

Antimicrobial stewardship (AMS) programs must evolve toward precision-based interventions. Patients with MetS are not only more susceptible to MDR colonization and infection; they also represent a high-risk group for resistance selection due to frequent and often inappropriate antibiotic exposure [110].

Furthermore, antimicrobial therapy in these individuals can exacerbate intestinal dysbiosis and contribute to worsening metabolic dysfunction [119].

AMS strategies should therefore include (1) empiric therapy guided by validated risk scores and local resistance patterns, (2) dose adjustments based on BMI and hepatic/renal function, (3) the preferential use of microbiota-sparing agents, and (4) the systematic application of therapeutic drug monitoring (TDM), particularly in ICU settings [112].

EHR-integrated metabolic alerts may facilitate pharmacist-led reviews of high-risk prescriptions in patients with elevated BMI or abnormal HOMA-IR values [116].

Multidisciplinary collaboration—including infectious disease specialists, clinical pharmacologists, internists, diabetologists, and nutritionists—is strongly recommended for managing these complex patients.

### 8.3. Public Health Implications and Preventive Policies

The syndemic impact of MetS and MDR infections on public health is substantial and requires comprehensive, multilayered intervention. Primary prevention of MetS—through promotion of physical activity, dietary education, and weight control—also serves as an indirect strategy for curbing antimicrobial resistance (AMR) by reducing the prevalence of metabolically vulnerable individuals at high risk for colonization and infection [120].

In Italy, the National Plan to Combat Antimicrobial Resistance (PNCAR) emphasizes the integration of prevention, surveillance, and stewardship, but does not yet explicitly address metabolic frailty as a risk factor [121].

Incorporating targeted recommendations for this emerging hospital subpopulation would enhance both the equity and translational impact of national policies.

Furthermore, the development of integrated surveillance systems—linking clinical, microbiological, and metabolic data—should be prioritized. Leveraging EHR capabilities and pharmacovigilance infrastructures could markedly improve risk stratification and guide timely interventions [116].

### 8.4. Community-Level Strategies for Managing the MetS–MDR Syndemic

Although most MDR containment efforts are concentrated in hospital settings, clinical experience reveals that many patients with MetS—particularly those with diabetes, MASLD, or severe obesity—receive repeated antibiotic courses in outpatient care for urinary, respiratory, or skin infections, thereby contributing to the community reservoir of resistance [121].

General practitioners and outpatient specialists must therefore be actively engaged in surveillance and containment strategies.

Territorial microbiological surveillance networks should be reinforced, especially in high-risk environments such as long-term care facilities and rehabilitation centers, where advanced age, metabolic dysfunction, and cumulative antibiotic exposure often coexist [122].

Recommended actions include the following:

- Systematic documentation of antibiotic histories in patients with MetS;
- Implementation of shared or delayed prescription models;
- Development of “metabolic decolonization” protocols involving dietary modulation, probiotics, and anti-inflammatory strategies;
- Inclusion of targeted vaccination programs (e.g., pneumococcal, influenza, herpes zoster) within infection prevention plans for metabolically at-risk patients, as endorsed by the ECDC and WHO [123,124,125].

## 9. Future Directions and Research Gaps

### 9.1. Knowledge Gaps in Immuno-Metabolic–Microbial Interactions

Despite growing interest in the interplay between metabolic syndrome (MetS) and infections caused by multidrug-resistant (MDR) pathogens, substantial knowledge gaps persist regarding the immunometabolic and microbial mechanisms that underlie their bidirectional relationship.

While chronic inflammation, metaflammation, and insulin resistance are well established as shared pathogenic drivers, the molecular pathways by which MDR infections may promote the onset or progression of MetS remain only partially elucidated [85].

To date, no longitudinal clinical studies have systematically tracked the metabolic trajectory of patients recovering from MDR infections, nor have they explored the differential risk of colonization and infection across various MetS phenotypes. This represents a critical limitation in understanding causality and refining clinical risk stratification.

Moreover, the roles of host genetic and epigenetic factors remain largely unexplored, despite their likely contribution to interindividual susceptibility and phenotypic heterogeneity. The integration of cross-omic platforms—including genomics, epigenomics, transcriptomics, metabolomics, and metagenomics—will be essential for identifying predictive molecular signatures, characterizing immunometabolic profiles at risk, and informing personalized preventive and therapeutic strategies.

In this context, identifying biomarkers capable of predicting MDR colonization, infection persistence, therapeutic response, or long-term metabolic remodeling is of particular clinical relevance, especially in patients with obesity, diabetes, or MASLD. Future research should also investigate the immunometabolic impact of chronic drug-resistant viral infections (e.g., HIV, HBV, CMV) as potential amplifiers of syndemic vulnerability.

### 9.2. Translational and Model-Based Studies

Experimental research on the MetS–MDR interface remains limited by the translational inadequacy of currently available preclinical models.

Most studies rely on obese or diabetic murine models infected with resistant bacterial strains. Although these models have generated valuable mechanistic insights, they fail to replicate the full complexity of human immunity, the metabolic diversity of patient phenotypes, and the distinctive architecture of the human gut microbiome [5].

There is an urgent need to develop more physiologically relevant experimental systems, including humanized models, organoid cultures, and ex vivo platforms, which enable integrated investigation of host–pathogen–microbiota interactions under metabolically compromised conditions.

Innovative approaches such as humanized mouse models, intestinal and hepatic organoids, and gut-on-chip systems hold great potential for reproducing the dynamic interplay between microbial ecosystems and host metabolism [126].

In parallel, computational systems biology and AI-driven modeling can simulate the impact of antibiotic pressure on the host resistome and identify novel pharmacological targets. These approaches are indispensable for advancing precision medicine across both infectious disease and metabolic care domains [127].

### 9.3. Innovation in Microbiome-Driven and Personalized Approaches

One of the most promising translational directions involves the development of microbiome-guided interventions aimed at preventing or mitigating the metabolic consequences of MDR infections [105].

Recent advances in metagenomic sequencing, microbial metabolomics, and microbial ecology enable the design of targeted strategies to modulate the gut microbiota, restore eubiosis, enhance insulin sensitivity, and suppress systemic inflammation [128].

Emerging therapies include precision-engineered probiotics and prebiotics designed to modulate specific metabolic and inflammatory pathways, as well as synthetic bacterial strains capable of outcompeting MDR organisms and curbing resistome expansion.

Standardized fecal microbiota transplantation (FMT) formulations may offer therapeutic benefits for patients with advanced dysbiosis and metabolically unstable phenotypes [129].

Concurrently, the identification of gut microbial signatures with diagnostic or prognostic utility is gaining traction, with potential applications in infection risk prediction and metabolic disease surveillance [74].

Together, these research directions underscore the need for a multidisciplinary and integrative research agenda that bridges microbiology, immunology, metabolism, and systems medicine. Addressing these gaps will be pivotal in transforming the conceptual framework of the MetS–MDR syndemic into actionable clinical strategies.

## 10. Conclusions

The interaction between metabolic syndrome (MetS) and infections caused by multidrug-resistant (MDR) pathogens defines a syndemic of increasing clinical and epidemiological relevance. This review has delineated a bidirectional and mechanistically plausible relationship: on the one hand, the immunologic and microbiota-related disturbances characteristic of MetS—including chronic low-grade inflammation, visceral adiposity, impaired innate and adaptive immunity, and gut dysbiosis—facilitate colonization and persistence of MDR organisms; on the other hand, severe or recurrent MDR infections may trigger systemic metabolic reprogramming, exacerbating insulin resistance, disrupting host–microbiota equilibrium, and perpetuating immunometabolic dysfunction.

Despite growing mechanistic insights, the current evidence base remains fragmented and largely associative. Key research gaps include the following:

(1) The absence of prospective, longitudinal studies to define the directionality and magnitude of the MetS–MDR relationship;
(2) The lack of validated biomarkers to predict infection risk or metabolic deterioration in this context;
(3) The limited translational utility of existing animal models, which fail to recapitulate the complexity of the human immune–metabolic–microbial interface;
(4) The need for clinically actionable, microbiota-based interventions capable of modulating both infectious and metabolic outcomes.

Addressing these challenges will require a coordinated and multidisciplinary research agenda. Priority areas include the following:

- Cohort studies stratified by metabolic phenotype to evaluate incidence, clinical outcomes, and longitudinal trajectories of MDR infections;
- Cross-omic analyses integrating genomics, epigenomics, transcriptomics, and metagenomics to identify susceptibility markers and immunometabolic signatures;
- The development of human-relevant experimental models—such as gut-on-chip systems, intestinal and hepatic organoids, and humanized mouse models—to simulate host–microbiota–pathogen interactions under metabolic stress;
- Clinical trials assessing the efficacy of microbiota-directed interventions, including targeted probiotics and standardized fecal microbiota transplantation, in high-risk metabolic populations;
- The integration of pharmacokinetic and pharmacodynamic data into antimicrobial stewardship protocols for patients with obesity, diabetes, or MASLD, to reduce therapeutic failure and resistance selection.

Acknowledging the MetS–MDR axis as a true syndemic carries implications that extend beyond the boundaries of infectious disease. It necessitates a paradigm shift in the prevention of chronic conditions, the formulation of antimicrobial policies, and the advancement of precision medicine. Future progress will hinge on the integration of metabolic, immunologic, and microbiological expertise, as well as the development of translational tools capable of transforming biological complexity into clinically actionable strategies.

## Figures and Tables

**Table 1:** Diagnostic criteria for metabolic syndrome according to the Joint Interim Statement (2009).

Diagnostic Criterion	Threshold/Definition	References
Abdominal Obesity	Increased waist circumference (sex- and ethnicity-specific cut-offs)	[25]
Hypertriglyceridemia	Serum triglycerides ≥150 mg/dL	[25]
Low HDL Cholesterol	<40 mg/dL in men; <50 mg/dL in women	[25]
Elevated Blood Pressure	≥130/85 mmHg or current use of antihypertensive medications	[25]

HDL: high-density lipoprotein.

**Table 2:** Molecular mechanisms of antimicrobial resistance in major pathogens.

Mechanism	Description	Common Pathogens	References
ESBL Production	Hydrolyzes extended-spectrum β-lactams	*E. coli*, *K. pneumoniae*	[55,56]
Carbapenemases (KPC, NDM, VIM, OXA)	Enzymatic degradation of carbapenems	*K. pneumoniae*, *P. aeruginosa*, *A. baumannii*	[55,56]
Ribosomal Methylation	Alters antibiotic binding sites	*Staphylococcus* spp., *Enterococcus* spp.	[55]
Efflux Pump Upregulation	Actively extrudes antibiotics	*P. aeruginosa*, *A. baumannii*	[55]
Porin Loss/Mutation	Reduces drug uptake	*K. pneumoniae*, *P. aeruginosa*	[55]
Target Modification	Alters PBPs or other drug targets	*S. aureus*, *E. faecium*	[55,56]
Horizontal Gene Transfer	Plasmid-mediated gene acquisition	Multiple species	[57]

ESBL: Extended-spectrum beta-lactamase, KPC: Klebsiella pneumoniae carbapenemase, NDM: New Delhi metallo-β-lactamase, VIM: Verona integron-encoded metallo-β-lactamase, OXA: Oxacillinase, PBPs: penicillin-binding proteins.

**Table 3:** Clinical and economic impact of major MDR pathogens.

Pathogen	Clinical Manifestations	Mortality/Economic Impact	References
*K. pneumoniae* (KPC-Kp)	Nosocomial infections: BSI, VAP	Hospital mortality >40%	[55,63]
*A. baumannii* (XDR)	ICU infections, biofilm formation	Mortality >50% in ventilated patients	[9,29]
*P. aeruginosa* (CRPA)	Infections in immunocompromised hosts	Increased length of stay, therapeutic failure	[10,64,65]
MRSA	Bacteremia, pneumonia, endocarditis	20–30% case-fatality in high-risk groups	[66,67]
CA-MRSA	Community-acquired infections	Enhanced virulence and spread	[68]
VRE (*E. faecium*)	Infections in immunocompromised patients	Requires expensive, toxic drugs	[69,70,71]
*K. pneumoniae* (NDM-1)	Nosocomial outbreak in NL	Cost USD ~804,263	[72]

KPC-Kp: *lebsiella pneumoniae* carbapenemase-producing *K. pneumoniae*, BSI: bloodstream infection, VAP: ventilator-associated pneumonia, XDR: extensively drug-resistant, ICU: intensive care unit, CRPA: carbapenem-resistant *Pseudomonas aeruginosa*, MRSA: methicillin-resistant *Staphylococcus aureus*, CA-MRSA: community-associated MRSA, VRE: vancomycin-resistant *Enterococcus*, NDM-1: New Delhi metallo-β-lactamase-1.

**Table 4:** Immunometabolic mechanisms linking metabolic syndrome to increased susceptibility to multidrug-resistant infections.

Mechanism	Description	Pathogens	References
Visceral Adiposity	Acts as a pro-inflammatory endocrine organ, producing TNF-α, IL-6, and leptin, with reduced adiponectin	*K. pneumoniae*, *A. baumannii*	[36,37,38,39]
Insulin Resistance	Impairs PI3K–AKT–mTOR pathway, reducing immune cell function (T cells, NK cells, macrophages)	Multiple MDR bacteria	[4,46]
Chronic Low-Grade Inflammation	Drives immune exhaustion via TLR-mediated activation and systemic cytokine release	*K. pneumoniae*, *P. aeruginosa*	[17,35,50]
Adaptive Immune Skewing	Increased Th1/Th17 polarization, reduced Treg function, and enhanced inflammation	*K. pneumoniae*, *A. baumannii*	[36,47]
Gut Dysbiosis	Promotes resistome expansion and epithelial barrier dysfunction	Enterobacteriaceae, *K. pneumoniae*	[50,74]

TNF-α: tumor necrosis factor-alpha; IL-6: interleukin-6; Pi3K-AKT-mTOR: phosphoinositide 3-kinase–protein kinase B–mammalian target of rapamycin; TLR: toll-like receptor; Th: T helper cell; Treg: regulatory T cell.

**Table 5:** Key genes, proteins, and pathways at the immunometabolic–antimicrobial resistance interface.

Gene/Protein	Function in Immunometabolism	Associated Pathway	Role in MDR Susceptibility	References
IRS-1	Transduces insulin signals to downstream effectors	IRS–PI3K–AKT–mTOR	Serine phosphorylation inhibits PI3K–AKT signaling, reducing immune cell function	[46]
AKT	Regulates cell survival, proliferation, and metabolism	PI3K–AKT–mTOR	Impaired activation compromises T-cell and macrophage responses	[46]
mTOR	Controls immune cell metabolism and growth	PI3K–AKT–mTOR	Dysregulation limits immune cell activation and proliferation	[46]
TLR4	Recognizes microbial PAMPs and endogenous ligands	TLR–NF-κB	Chronic activation promotes metaflammation, immune exhaustion	[17]
IL-6	Mediator of systemic inflammation	NF-κB pathway	Elevates systemic inflammation, impairs pathogen clearance	[17,39]
TNF-α	Pro-inflammatory cytokine	NF-κB pathway	Contributes to chronic inflammation and immune dysregulation	[39]
Adiponectin	Anti-inflammatory adipokine	Metabolic regulation	Low levels worsen insulin resistance and inflammatory status	[41]

IRS: insulin receptor substrate, PI3K: phosphoinositide 3-kinase, AKT: protein kinase B, mTOR: mammalian target of rapamycin, TLR: toll-like receptor, NF-κB: nuclear factor kappa-light-chain-enhancer of activated B cells, PAMPs: pathogen-associated molecular patterns, IL-6: interleukin-6, TNF-α: tumor necrosis factor-alpha.

**Table 6:** Metabolic syndrome as a predictor of clinical outcomes in MDR infections.

Study Context	MetS Component(s)	Adverse Outcome(s)	References
ICU patients with MDR *A. baumannii*	Obesity, T2DM	Increased mortality, longer ICU stay, mechanical ventilation	[83,84]
Patients with KPC-Kp bacteremia	Obesity (BMI > 30)	Higher in-hospital mortality, treatment failure	[82]
Spanish multicenter cohort	Diabetes	Higher 30-day mortality, recurrence	[85]
General ICU population with sepsis	Obesity	Increased mortality, worse infection control	[86]

T2DM type 2 diabetes mellitus; KPC-Kp: *Klebsiella pneumoniae* carbapenemase-producing strain; ICU: intensive care unit; BMI: body mass index.

**Table 7:** Pathogenetic interactions between MetS and MDR (bidirectionality).

Factor	MetS Impact on Infection	Impact of Infection on MetS	References
Insulin Resistance	Impaired phagocytic activity and pathogen clearance	Aggravated via TLR4-mediated inflammation	[27,42,98]
Intestinal Dysbiosis	Promotes MDR colonization and translocation	Worsened by antibiotic-induced microbiota disruption	[54,55,102]
Chronic Inflammation	Enhances MDR pathogen virulence and persistence	Amplified by recurrent or unresolved infections	[6,45,95]
Immune Alterations	Skews toward Th1/Th17 responses, impacting host defense	Triggered and sustained by chronic infectious stimuli	[23,52,53]

MetS: metabolic syndrome, TLR: toll-like receptor, MDR: multidrug resistance, Th: T-helper cell.

**Table 8:** Impact of metabolic syndrome on antimicrobial pharmacokinetics and pharmacodynamics.

Factor in MetS	Affected PK/PD Parameter	Antimicrobial Class	Clinical Implication	References
Visceral Adiposity	Increased volume of distribution	β-lactams, aminoglycosides (hydrophilic)	Risk of subtherapeutic levels	[104,107]
Insulin Resistance and MASLD	Altered hepatic metabolism	Fluoroquinolones, macrolides (lipophilic)	Variable drug exposure, potential toxicity	[104,107,108]
Obesity	Need for adjusted dosing weight	Aminoglycosides, colistin	Risk of nephrotoxicity/ototoxicity if misdosed	[109,110]
Hepatic Inflammation	CYP450 dysregulation	CYP450-metabolized agents	Requires hepatic function assessment	[104,108]
Critical Illness	PK variability	Multiple antibiotic classes	Benefit from therapeutic drug monitoring (TDM)	[111]

PK: pharmacokinetics, PD: pharmacodynamics, MetS: metabolic syndrome, MASLD: metabolic dysfunction-associated steatotic liver disease, CYP450: cytochrome P450, TDM: therapeutic drug monitoring.

**Table 9:** Strategic components of personalized management in MetS–MDR patients.

Clinical Domain	Personalized Strategy in MetS Context	Rationale and Therapeutic Target	References
Antimicrobial Dosing	Adjust dosing based on BMI and hepatic function	Accounts for altered volume of distribution and metabolism in obesity/MASLD	[106,107,112]
Therapeutic Drug Monitoring	Apply TDM for β-lactams, aminoglycosides, and colistin	Addresses PK variability, reduces toxicity, and ensures efficacy	[110]
Microbiota Restoration	FMT; targeted probiotics and prebiotics	Corrects dysbiosis, limits resistome spread	[54,92,101]
Immunometabolic Modulation	Use polyphenols, SCFAs, and immune checkpoint inhibitors	Mitigates chronic inflammation, enhances host immunity	[93,114]
Multidisciplinary Care	Coordinate ID–Internal Medicine–Pharmacology–Nutrition	Facilitates integrated care for complex host–pathogen dynamics	[4,112]

MetS: metabolic syndrome, BMI: body mass index, MASLD: metabolic dysfunction-associated steatotic liver disease, TDP: therapeutic drug monitoring, PK: pharmacokinetics, SCFA: short-chain fatty acids, ID: infectious disease.

## Data Availability

No datasets were generated or analyzed for this study.
